# Ubiquitin-dependent and independent roles of SUMO in proteostasis

**DOI:** 10.1152/ajpcell.00091.2016

**Published:** 2016-06-22

**Authors:** Frauke Liebelt, Alfred C. O. Vertegaal

**Affiliations:** Department of Molecular Cell Biology, Leiden University Medical Center, Leiden, the Netherlands

**Keywords:** proteostasis, SUMO, ubiquitin, SUMO-targeted ubiquitin ligase, neurodegenerative diseases, protein aggregations

## Abstract

Cellular proteomes are continuously undergoing alterations as a result of new production of proteins, protein folding, and degradation of proteins. The proper equilibrium of these processes is known as proteostasis, implying that proteomes are in homeostasis. Stress conditions can affect proteostasis due to the accumulation of misfolded proteins as a result of overloading the degradation machinery. Proteostasis is affected in neurodegenerative diseases like Alzheimer's disease, Parkinson's disease, and multiple polyglutamine disorders including Huntington's disease. Owing to a lack of proteostasis, neuronal cells build up toxic protein aggregates in these diseases. Here, we review the role of the ubiquitin-like posttranslational modification SUMO in proteostasis. SUMO alone contributes to protein homeostasis by influencing protein signaling or solubility. However, the main contribution of SUMO to proteostasis is the ability to cooperate with, complement, and balance the ubiquitin-proteasome system at multiple levels. We discuss the identification of enzymes involved in the interplay between SUMO and ubiquitin, exploring the complexity of this crosstalk which regulates proteostasis. These enzymes include SUMO-targeted ubiquitin ligases and ubiquitin proteases counteracting these ligases. Additionally, we review the role of SUMO in brain-related diseases, where SUMO is primarily investigated because of its role during formation of aggregates, either independently or in cooperation with ubiquitin. Detailed understanding of the role of SUMO in these diseases could lead to novel treatment options.

proteostasis refers to the ability of the cell to adapt to stresses and protect the normal function of individual proteins in the proteome. Proteins are the key workforce of the cell; therefore, keeping them in a balanced equilibrium by maintaining their folding and regulating their expression and degradation is critical for proper functioning of the cell (7, 50). Multiple pathways are involved in the control of this pivotal task. Molecular chaperones assist in protein folding of newly synthesized or misfolded proteins, and their expression can be increased upon proteotoxic stresses, where they contribute to process the overload of unfolded or misfolded proteins (56). Misfolded proteins are degraded by the ubiquitin proteasome system (UPS) via the attachment of the posttranslational modifier ubiquitin, which targets proteins to the proteasome for destruction. The proteasome is a multisubunit protein complex, which is highly conserved in eukaryotes, archaea, and even present in some eubacteria, indicating its importance (37). The proteasome is able to degrade misfolded proteins and recycle amino acids which can be reused for protein synthesis (47). Controlled degradation of proteins is important to prevent toxic protein aggregates and in the context of signaling (7). Chaperones and the UPS are part of a complex proteostasis network (PN) (50). Here we review how small ubiquitin-like modifiers (SUMOs) contribute to proteostasis.

SUMOs are members of the family of ubiquitin-like proteins (UBLs) (31, 33). Common hallmarks of UBLs are the presence of an ubiquitin fold, a COOH-terminal Gly-Gly motif, and an enzymatic cascade which is needed for the conjugation to their substrates (Fig. 1). Although different UBLs employ distinct sets of enzymes, the mature form of SUMO is first activated by the heterodimeric SUMO activating enzyme (E1) composed of SAE1 and SAE2 (also known as UBA2), which, in an ATP-dependent two-step reaction, forms a thioester-bond with SUMO. After activation, SUMO is transferred to the single SUMO E2 conjugation enzyme UBC9 (also known as UBE2I). UBC9 is not only able to provide the activated SUMO, but is additionally involved in substrate binding and specificity. UBC9 has binding affinity to the SUMOylation consensus motif within the substrate, which is defined by the amino acids composition ΨKXE, where Ψ stands for a large hydrophobic residue and X stands for any amino acid. Some SUMOylation targets harboring a consensus motif can be SUMOylated efficiently in vitro by the addition of the E1 and E2 only.

**Fig. 1.**
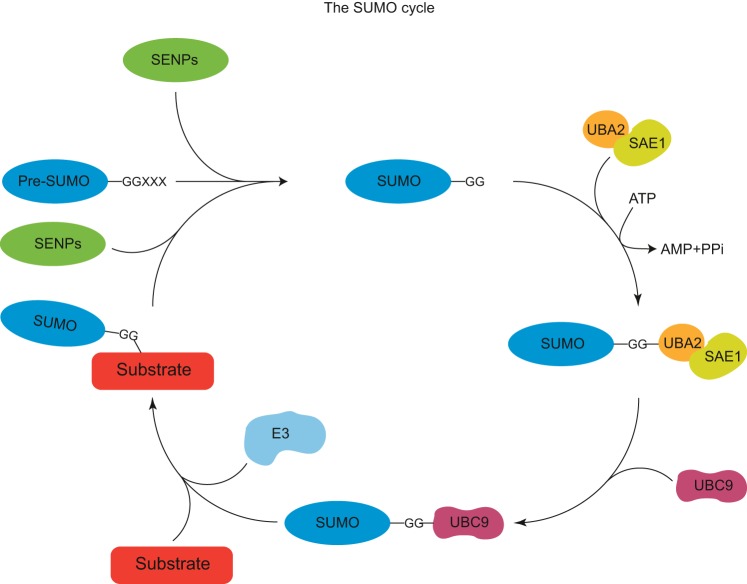
The SUMO cycle. The SUMO precursor protein is processed by SUMO proteases (SENPs) to expose the COOH-terminal GG motif. In an ATP-dependent step, the heterodimeric SUMO E1-activating enzyme, consisting of SAE1 and UBA2, forms a thioester bond with SUMO. SUMO is then transferred to UBC9, the SUMO E2 conjugation enzyme, which is responsible for the isopeptide bond formation between the COOH-terminal GG motif and the target lysine residue of the substrate. This step is enhanced by SUMO E3 ligases. The modification is reversible as SENPs can remove the SUMO moiety from the substrate.

In vivo the activity of SUMO E3 ligases is necessary for efficient conjugation of SUMO to its substrates. In yeast, four E3 ligases are identified so far, Siz1, Siz2, Mms21, and Zip3 (15, 58, 116, 148). In mammals, an ability to catalyze the conjugation of SUMO to its target proteins is demonstrated for proteins belonging to the Siz/protein inhibitor of activated STAT (PIAS) family, the nucleoporin RanBP2 and ZNF451. Several other proteins are proposed as SUMO E3 ligases, including the human polycomb protein Pc2/CBX4 (60), the topoisomerase I-binding protein Topors (132), the tumor suppressor p14 Arf (114, 137), the RWD-containing SUMOylation enhancer RSUME (13), the small G protein which is specifically expressed in the striatum Rhes (111), and the Fanconi anemia protein SLX4 (38, 95). SUMO E3 ligases can strengthen the interaction between UBC9 and the target substrate, or position UBC9 in a way that is beneficial for the transfer of SUMO to the substrate, thereby stimulating SUMOylation.

The conjugation of SUMO is a tightly regulated and dynamic process which can be reversed by SUMO-specific proteases. Ubiquitin-like specific proteases Ulp1 and Ulp2 are responsible for deSUMOylation of proteins of *Saccharomyces cerevisiae*. Ulp1 also processes the SUMO precursor protein at its COOH terminus, which is needed for the conjugation of SUMO to a target protein. Two protein families act as SUMO proteases in mammals. Members of the Ulp/sentrin-specific protease (SENP) family process SUMO precursors and deSUMOylate conjugated targets. Members of the second family of SUMO proteases are desumoylating isopeptidase (DeSI) 1 and 2, which are primarily used for deconjugation of SUMO (reviewed in refs. 25, 43, 48). Additionally, ubiquitin-specific protease-like 1 (USPL1) has been identified as a SUMO protease (106).

In *S. cerevisiae*, only a single SUMO isoform has been identified, Smt3. In mammals, the SUMO family consists of three members, SUMO1, 2, and 3, encoded by three different genes. Mature SUMO2 and 3 share 97% amino acid sequence identity and are so far not distinguishable through specific antibodies and therefore often referred to as SUMO2/3. The mammalian SUMO2 and SUMO3 as well as the yeast Smt3 can form polySUMO chains owing to their intrinsic ΨKXE-type SUMO conjugation consensus motifs. Mammalian SUMO1 shares only 47% amino acid sequence with SUMO2 and 3. Because of the absence of the intrinsic ΨKXE-type SUMO consensus motif, SUMO1 is not efficiently incorporated in SUMO chains but can be used to terminate a SUMO2/3 chain or can be conjugated to a target substrate as single moiety (84). However, more recently, it was found that SUMO1 contains an inverted SUMOylation motif, enabling SUMO polymerization to some extent (83). SUMO1 and SUMO2/3 share substrates but also target distinct sets of proteins (10). Interestingly, SUMO1-deficient mice are viable because of the ability of SUMO2 and 3 to compensate for SUMO1 loss (146). Knockout of SUMO2 is embryonically lethal, while mice lacking SUMO3 are viable. This observation can be explained by the fact that SUMO2 is the predominantly expressed isoform (130).

SUMOylation is a modification with numerous different functions, as it can activate or repress its target proteins, it can alter protein-protein interactions or result in changes in subcellular localization (31). Interestingly, SUMOylation levels are dynamically regulated by various stresses, linking SUMO to regulation of cellular homeostasis. In this review, we will focus on the SUMO literature concentrating on the importance of SUMOylation in the homeostasis of proteins either directly or in cooperation with ubiquitin.

## SUMO Is Involved in Proteostasis via Crosstalk With Ubiquitin

The ubiquitin-proteasome system is the central mechanism of proteostasis and responsible for the regulated degradation of proteins and recycling of amino acids. SUMO is highly connected to this process as detailed below.

### SUMO and ubiquitin can modify the same lysine residue in a protein.

Recent advances in high-resolution mass spectrometry (MS) have enabled large-scale identification of SUMO acceptor lysines and ubiquitin acceptor lysines. The obtained results indicate that almost a quarter of SUMO-acceptor lysines are also used for ubiquitin conjugation, pointing towards extensive crosstalk between these modifications (44, 117). This kind of competition was first found in 1998 when Hay and coworkers showed that the inhibitor of NF-κB, IκB-α, could be SUMOylated at the same lysine residue that is also used for ubiquitination (22).

The modification of SUMO and ubiquitin of the same acceptor lysine can also act sequentially. This is illustrated by the serine hydroxymethyltransferase SHMT1, and the regulatory subunit of the IκB kinase, NEMO. For both proteins it was shown that SUMOylation stimulated their translocation to, and retention within, the nucleus. After cleavage of the SUMO moiety in the nucleus, the same lysine residue could be modified by ubiquitin, which stimulated subsequent export to the cytoplasm (4, 52). These examples illustrate that there is a tightly regulated balance, in time and space, between ubiquitin and SUMO targeting the same lysine within a protein, which determines function, localization, or stability.

The proliferating cell nuclear antigen (PCNA) is an intriguing example of the cooperative effect of SUMO and ubiquitin on the same acceptor lysine. PCNA encircles the DNA as a sliding clamp and accompanies processing DNA polymerases, additionally providing an important interaction stage for proteins involved in DNA repair (reviewed by ref. 59). In yeast, encountering a DNA lesion during replication leads to either mono- or polyubiquitination of PCNA at the lysine residue K164. While monoubiquitination stimulates the recruitment of translesion polymerases η and ζ to facilitate the error-prone DNA damage tolerance pathway, polyubiquitination of K164 promotes the error-free damage avoidance pathway (51, 110). SUMOylation of PCNA also targets K164. In contrast to the ubiquitination of PCNA, its modification by SUMO appears to be DNA damage independent but reliant on S-phase. In addition to the major SUMOylation site at K164, K127 is a minor SUMOylation site, increasingly used for modification after K164R mutagenesis (51). SUMOylated PCNA recruits the helicase SRS2, which was shown to promote the DNA damage tolerance pathway and inhibits unscheduled homologous recombination in multiple studies (12, 97, 127). SUMOylation of PCNA therefore promotes the ubiquitin-dependent tolerance pathway during S-phase, in case the replication fork encounters a lesion. Even though both modifications target the same lysine, the crosstalk is successive rather than competitive, comparable to the above mentioned regulation of NEMO and SHMT1.

The idea that SUMO and ubiquitin can modify the same acceptor lysine does not necessarily result in the need for successive modifications or competition. Ubiquitin and especially SUMO are only conjugated to a small subset of a given protein, making it possible for both modifiers to be present on the same lysine at the same time but in different subpopulations of the target proteins.

### SUMO-targeted ubiquitin ligases.

In addition to the model of exclusive occupation by either ubiquitin or SUMO at one acceptor site of a protein, there are indications that both modifications can form a hybrid chain. Site-specific mass spectrometry approaches have identified SUMOylation sites on endogenous ubiquitin at multiple positions (44). Endogenous SUMO can also be modified by ubiquitin, preferably at the K11 position, which lies within a SUMOylation consensus site (19). The establishment of SUMO-ubiquitin chains is catalyzed by a specific group of ubiquitin ligases, specifically targeting SUMOylated protein.

SUMOylation of a protein can directly serve as recognition signal for SUMO-targeted ubiquitin ligases (STUbLs) (99, 118, 124). The ability of STUbLs to recognize SUMOylated proteins is mediated by their SUMO-interaction motifs (SIMs) and a RING domain, which enables them to bind to SUMOylated proteins and an E2 ubiquitin-conjugation enzyme, respectively. The presence of multiple SIMs, within the identified STUbLs, determines their preference for substrates with SUMO chains (118, 124). In *S. cerevisiae*, three potential STUbLs have been identified, Uls1, Slx5/Slx8, and Rad18. The ubiquitin ligase for SUMOylated proteins (ULS) 1 and the heterodimeric STUbL Slx5/Slx8 are responsible for the proteolytic control of SUMOylated proteins in yeast (124, 139). Notably, Slx5/Slx8 does not necessarily require SUMOylation of its targets, but its activity is stimulated by SUMOylation. This stimulation is likely explained by an enhancement of target-enzyme interaction via SIMs on the NH_2_ terminus of Slx5 (134, 140). The ubiquitin ligase Rad18, which is responsible for the ubiquitination of the sliding clamp PCNA, is stimulated by the SUMOylation of PCNA in yeast. Human Rad18, however, does not show STUbL activity due to the lack of SIM motifs (98).

In humans, the E3 ubiquitin ligase RNF4 targets SUMO conjugates. RNF4 contains four putative SIMs, which show binding affinity not only to SUMO2 but also to SUMO1. However, RNF4 prefers to target proteins that are modified by SUMO chains of at least three SUMO moieties (118). RNF4 regulates substrates involved in a multitude of pathways, including kinetochore assembly (87), cell survival upon hypoxic stress (125), mitogen-activated protein (MAP) kinase signaling (39), transcriptional responses to heat shock (82), ion transport (2), and the DNA damage response (91). The most extensively studied substrate of RNF4 is the promyelocytic leukemia protein (PML). SUMOylation of PML is stimulated after treatment with arsenic trioxide (ATO), and SUMOylated PML is targeted by RNF4 for ubiquitination and subsequent proteasomal degradation (71, 118, 133). Interestingly, it was shown that PML ubiquitination by RNF4 took place on PML itself but also on the SUMO moiety attached to PML, indicating the formation of a hybrid SUMO-ubiquitin chain (118). PML was not only found to be SUMOylated and targeted by RNF4, but was additionally indicated to cooperate with RNF4 in the degradation of misfolded proteins in the nucleus. The polyQ pathogenic protein ataxin 1 (Atxn1 82Q), which is the causative, aggregation prone protein for a type of spinocerebellar ataxia (SCA), was shown to be SUMOylated. This SUMOylation was enhanced by PML and subsequently Atxn1 82Q could be targeted by RNF4 for ubiquitination and proteasomal degradation. RNF4 was further shown to reduce other misfolded proteins in the nucleus, including the polyQ huntingtin (40). A general role of RNF4 in the degradation of misfolded proteins in the nucleus was proposed on the basis of this finding, highlighting the possibility of a therapeutic effect by manipulating SUMOylation in neurodegenerative diseases, which will be discussed in more detail later.

Recently, the transcription factor and oncogene c-Myc was shown to be targeted by SUMO and ubiquitin modifications, regulated by RNF4. These modifications led to the rapid degradation of c-Myc in a proteasome-dependent manner. The authors showed that mutagenesis of all SUMOylation sites which were identified by mass spectrometry did not lead to a reduced SUMOylation of c-Myc, suggesting that the attachment of SUMO to a lysine residue might be promiscuous within the protein (36). These results are compatible with the idea that SUMO and ubiquitin modifications might be arbitrary in proteins that are targeted for degradation and the exact location of the modification plays a subordinate role here.

A second human STUbL is the ubiquitin ligase RNF111, also called Arkadia. RNF111 with its three putative SIMs was identified in a bioinformatic screen for SIM-containing proteins (113). RNF111 has previously been implicated in the TGF-β signaling pathway, where it is responsible for the ubiquitination and degradation of the negative regulators SMAD7, c-Ski, and SnoN.

As multiple factors involved in the TGF-β pathway are SUMOylated, including TGF-β receptor (61), Smad3 (53), Smad4 (73), and Axin (62), the STUbL activity of RNF111 might be involved in the regulation of this pathway, but this remains largely speculative. One study showed that the SIMs of RNF111 are not needed for the degradation of c-Ski and SnoN (28). Intriguingly, like RNF4, RNF111 was shown to regulate the proteasomal degradation of SUMOylated PML upon ATO treatment, suggesting that both STUbLs are involved and important in the regulation of the PML nuclear bodies (28).

### Hybrid SUMO-ubiquitin chains.

The activity of STUbLs and the establishment of SUMO-ubiquitin chains on a protein can either act as a recruitment signal or can target proteins to the proteasome. Both processes seem to be especially important for the regulation of the chromatin environment (32, 128, 144).

The first identified hybrid SUMO-ubiquitin chain receptor involved in mediating recruitment of proteins to the chromatin is RAP80, a component of the BRCA1-A complex. RAP80 carries a tandem ubiquitin interaction motif (UIM) and a SIM, and it consistently shows preferential affinity for hybrid chains. The establishment of hybrid chains at DNA damage sites, through the activity of the SUMO-targeted ubiquitin ligase RNF4, recruits RAP80 and the BRCA1-A complex to DNA lesions (41). In addition, the proteasomal subunit S5a/RPN10, which has protease activity, bears UIM and SIM motifs and it is intriguing to think that this protein could be responsible for the docking of hybrid SUMO-ubiquitin chains at the proteasome (42).

Furthermore, the targeting of a substrate to the proteasome via STUbL catalyzed SUMO-ubiquitin chains might possibly involve the recruitment of proteins mediating this process. A likely candidate is the AAA-ATPase p97. P97, or its yeast homologue CDC48, is able to extract ubiquitinated protein from the chromatin (reviewed by refs. 6, 85). Interestingly, CDC48 and its cofactors Ufd1 and Npl4 cooperate with SUMO at the chromatin. Yeast Ufd1 exhibits a COOH-terminal SIM motif, which enables a noncovalent interaction between SUMO and the CDC48-Ufd1-Npl4 complex (90). Yeast Ufd1 mutants displayed subnuclear foci of accumulated SUMO-conjugates, indicating a role of Ufd1 in the degradation of SUMOylated proteins (64, 90). Furthermore, the CDC48-Ufd1-Npl4 complex was shown to physically interact with STUbLs (64). These observations strengthened the idea that STUbL-induced proteasomal targeting of SUMO-conjugates could be facilitated by the segregase activity of CDC48, especially within the chromatin context where proteins might be “stuck” to the DNA and specific forces are needed to extract them. A site-specific mass spectrometry approach making use of genetic mutants of either the yeast STUbL subunit Slx8 or the CDC48 cofactor Ufd1 identified a subset of proteins which were coordinately regulated by both. These proteins were associated with centrosomes and telomeres, showing the importance of CDC48 segregase activity on proteins bound to DNA (65). In mammalian cells, the Fanconi anemia complex FANCI/FANCD2 (ID complex) is a target of RNF4-triggered extraction by p97. SUMOylation of the ID complex upon treatment with agents causing replication fork stalling induced its RNF4-dependent ubiquitination. P97 together with its cofactor DVC1 was subsequently responsible for the removal of the ubiquitinated ID complex and therefore regulates the amount of activated FANCI and FANCD2 on the chromatin. Direct targeting of SUMOylated proteins by yeast CDC48 without the contribution of ubiquitin was demonstrated by Jentsch and coworkers (11). They showed that the SUMOylated DNA repair factor RAD52 physically interacted with CDC48, leading to displacement from the chromatin of RAD52 together with its binding partner RAD51. This CDC48 effect was dependent on RAD52 SUMOylation as well as the SIM motif of Ufd1 and independent of ubiquitin, showing that CDC48 is able to directly target SUMOylated proteins (11).

### SUMO deubiquitinases act on SUMO-ubiquitin chains.

Signal transduction by hybrid SUMO-ubiquitin chains can be counteracted by a ubiquitin-protease with the ability to reverse the action of STUbLs. The deubiquitinating enzyme USP11 was identified as a binding partner of RNF4 through mass spectrometry analysis. In vitro experiments showed that USP11 was able to deubiquitinate hybrid SUMO2-ubiquitin chains produced by RNF4. USP11 could counteract RNF4 under normal growth conditions and within the DNA damage response (45, 138). Multiple studies showed the involvement of RNF4 and USP11 in the DNA damage response and reflect the importance of reversible ubiquitination of SUMOylated proteins (32, 103, 128, 136, 144). The concept of a deubiquitinase that specifically targets hybrid SUMO-ubiquitin chains was further demonstrated by the identification of USP7 as SUMO deubiquitinase (SDUB) involved in DNA replication. USP7 was shown to establish the earlier observed SUMO-rich, ubiquitin-poor surroundings of replisomes by limiting ubiquitination of SUMOylated proteins, consistently hindering their clearance from the replication site. The authors also demonstrated that the clearance of the ubiquitinated SUMO conjugates was dependent on the action of the AAA-ATPase p97 (72).

Taken together, the ability of cells to form hybrid chains between ubiquitin and SUMO, possibly branched or including different SUMO family member, opens up numerous possibilities of cooperative SUMO and ubiquitin signaling. Specific proteins are needed for the catalysis, recognition, and destabilization of hybrid chains, and only a few have been identified so far.

### Ubiquitin-stimulated SUMOylation could be involved in stress responses.

Multiple studies showed that, upon inhibition of the proteasome, ubiquitinated proteins as well as SUMO2/3-conjugated proteins accumulated (101, 119). The inability to degrade a SUMOylated protein via the ubiquitin-proteasome pathway after proteasome inhibition could be explanatory for the increase of SUMO conjugates. This explanation, however, implies a simultaneous increase of SUMO and ubiquitin conjugates, whereas it was shown that the increase of SUMO2/3 targets is delayed compared with ubiquitin accumulation (101, 119). A possible explanation for this observation is the direct stimulation of SUMOylation after proteasome inhibition. This possibility is supported by the finding that the accumulation of SUMO2/3-modified proteins after proteasome inhibition was likely triggered by newly synthesized misfolded proteins which failed to be degraded (119). Interestingly, the authors of this paper showed that ubiquitin associated with the SUMO2/3 conjugates first decreased after proteasomal inhibition and then increased again with an enrichment of K63-linked ubiquitin chains, which were previously shown to be involved in the regulation of misfolded proteins. Therefore the accumulation of SUMO substrates in the cell upon proteasome inhibition is possibly only partly a result of the stabilization of SUMO substrates and might be explained by an active SUMOylation response upon misfolded protein stress. This hypothesis is also supported by the finding that a significant amount of proteins that are SUMOylated upon heat shock, a stress which induces unfolding of proteins, overlap with proteins that were SUMO modified after proteasome inhibition (44, 119). These data suggest a possible ubiquitination-stimulated SUMOylation of a subset of proteins.

## SUMOylation Regulates Proteostasis at the Chromatin Level

Transcription factors and chromatin bound proteins are common targets of SUMOylation, and the modification of these factors results in either a repressive effect on gene expression or a transcriptional activation (96).

Investigating the positioning and functional consequences of SUMOylated chromatin bound proteins, revealed that SUMO conjugates were enriched on active chromatin and at promoters of histones and genes encoding protein biogenesis components as well as tRNA and rRNA genes. SUMOylation was shown to have a repressive function in all cases, because knockdown of UBC9 resulted in an increased transcription of those genes (89). Additionally, SUMO plays a role in global transcriptional downregulation in response to DNA damage (46). These findings do not only indicate an overall repressive effect of SUMOylation on transcription factors and chromatin bound proteins, but also suggests that SUMOylation regulates protein homeostasis already at the transcriptional level.

The important role of SUMO in global transcriptional regulation is also reflected by the finding that the SUMO landscape at the chromatin is drastically changed upon heat shock (HS). HS increased the overall occurrence of SUMO at active chromatin, and SUMO was specifically enriched at promoters and lost from intergenic regions. Target genes of HS-induced chromatin-associated SUMO conjugates encode proteins involved in gene expression and posttranslational modification of RNA (92, 107).

Data on the transcriptional consequences of the HS-induced SUMO recruitment to promoter regions indicate target-gene specific effects of SUMOylation. Palvimo and coworkers found that impaired SUMOylation resulted in higher transcriptional activation of genes responsive to HS, whereas HS-induced repression was lost (92). They specifically showed that SUMO was enriched at the promoters of the heat shock protein (HSP) gene cluster upon HS and limited hyperactivation of the gene cluster, thus adapting a repressive role at those promoters (92). In contrast, another study proposed that SUMOylation of chromatin-bound proteins upon HS is required for the maximal transcription of genes involved in cell death and survival while SUMO has an repressive effect on genes involved in gene expression and cell cycle progression. They propose a model in which SUMOylation might either act as a molecular glue to facilitate stability of protein complexes at the promoter of genes important for the stress response, or that SUMOylation might increase solubility of proteins at the chromatin and block aggregation, thereby facilitating a correct folding and functioning of chromatin-bound proteins (107). The differences between the studies can possibly be explained by the usage of different cell lines and differences in analytical tools. Although these findings contributed to the overall understanding of SUMOylation activity at the chromatin level, the identity of the SUMO-targeted proteins at the different target genes remains to be established at large.

Interestingly, Gardner and coworkers recently showed that the conserved transcriptional corepressor Cyc8 is SUMOylated upon hyperosmotic stress in yeast (94). Loss of Cyc8 SUMOylation leads to cytoplasmic inclusion formation of this protein. Thus, SUMO acts to maintain the proper function of Cyc8 during hyperosmotic stress (94). This provides an interesting example of the role of SUMO during stress to maintain the solubility and thereby the function of these proteins. SUMO could regulate other target proteins during stress in a similar manner to block their aggregation, thereby maintaining their functionality.

## SUMOylation Regulates Protein Aggregation in Neurodegenerative Disease

The direct and indirect roles of SUMO in proteostasis are increasingly of interest in diseases characterized by a deregulation of proteostasis, such as neurodegenerative diseases. A hallmark of neurodegenerative diseases is the progressive loss of neurons. While neurodegenerative diseases are clinically diverse, depending on the group of neurons affected, these diseases share some underlying impairments. Common compromised processes are mitochondrial function upon oxidative stress, RNA function and metabolism, the UPS system, and the altered solubility of specific disease-associated proteins (14, 105, 126). The UPS system is being extensively investigated in this context, and failure of proper protein degradation plays an important part in the pathogenesis of neurodegenerative diseases (149). SUMOylation is involved in multiple neurodegenerative diseases, where key proteins were found to be SUMO modified. Together with the ubiquitin system, or independently, SUMO affects protein-protein interaction, protein activity, and most importantly, stability and solubility of several disease-associated proteins (Table 1) (67). Unraveling the influence of SUMO in these diseases can potentially lead to the development of novel drugs and treatment strategies.

**Table 1.** Involvement of SUMO in the formation of disease-associated protein aggregates

Disease	Affected Protein	Consequence of Aggregation	Influence of SUMO	Proposed Net Effect of SUMO on Cell Viability	Reference
Alzheimer's disease	Amyloid-β	Unknown if causal or consequential	Increases Aβ secretion	Negative	Dorval et al., 2007 (24)
			Reduces aggregation	Positive	Zhang and Sarge, 2008 (147)
			Reduces Aβ secretion	Positive	Li et al., 2003 (78)
			Increases Aβ secretion	Negative	Yun et al., 2013 (145)
	Tau	Cytotoxic	Reduces solubility and inhibits degradation	Positive	Luo et al., 2014 (80)
Parkinson's disease	α-Synuclein	Cytotoxic	Increases solubility	Positive	Krumova et al., 2011 (66)
			Increases solubility	Positive	Abeywardana et al., 2015 (1)
	DJ-1	Associated with inclusions	Incorrect SUMOylation decreases solubility	Positive and negative	Shinbo et al., 2006 (108)
					
Huntington's disease	mHTT	Cytoprotective	Associates with aggregates	Negative	O'Rourke et al., 2013 (93)
			Stabilizes pathogenic fragment of HTT and reduces aggregation	Negative	Steffan et al., 2004 (109)
					
Spinobulbar muscular atrophy	Androgen receptor	Cytotoxic	Increases solubility	Positive	Mukherjee et al., 2009 (86)
					
Spinocerebellar ataxin type 1	Ataxin-1	Cytotoxic	Reduces aggregation	Positive	Guo et al., 2014 (40)
					
Spinocerebellar ataxin type7	Ataxin-7	Cytotoxic	Increases solubility	Positive	Janer et al., 2010 (55)
					
Dentatorubral-pallidoluysian atrophy	Atrophin 1	Cytotoxic	Reduces aggregation	Positive	Terashima et al., 2002 (120)
					
Familiar amytrophic lateral sclerosis	SOD1	Cytotoxic	Increases aggregation	Negative	Fei et al., 2006 (29)

### Alzheimer's disease.

The molecular characteristics of Alzheimer's disease (AD) are extracellular senile plaques and intracellular neurofibrillary tangles, composed of aggregated amyloid-β (Aβ) peptides and aggregates of the microtubule-associated protein tau, respectively (70, 121). The particular molecular causes of AD are still under investigation, but it is clear that AD is a complex, multifactorial disease.

SUMOylation of the amyloid precursor protein (APP) was reported at two lysine residues, which were close to the cleavage site important for the production of Aβ. Interestingly, in the Swedish early-onset familiar form of AD, one of the identified SUMOylation sites is mutated, which indicates a potential role of SUMOylation in AD. Additionally, multiple studies reported changes in Aβ processing or production, because of manipulation of the SUMO pathway. However, the observed results are conflicting. While the RNAi knockdown of SUMO1 and SUMO2 does not affect Aβ production in HEK293 cells, it was shown that the SUMOylation of APP in HeLa cells negatively influenced the aggregation of Aβ (24, 147). Also, the effect of overexpressing SUMO3 was shown to either reduce (78) or increase (24) the production of Aβ. Dissimilarities can be explained by different experimental methods, partial redundancy of SUMO isoforms, indirect effects of manipulating the SUMO pathway, or overexpression effects. Nevertheless, involvement of SUMOylation in Aβ regulation is worth investigating in further detail.

Neurofibrillary tangles, the second hallmark of AD, are aggregates of the hyperphosphorylated form of the microtubule associated, natively unfolded protein tau, which in its nonhyperphosphorylated form is promoting tubulin stability (3, 54). The SUMOylation of tau at lysine residue K340 promoted its hyperphosphorylation, resulting in a reduced ubiquitination, therefore potentially stabilizing tau and promoting aggregation. In line with these findings, inhibiting the proteasome led to an increase in ubiquitination of tau and a decrease in SUMOylation, suggesting that crosstalk between SUMOylation, ubiquitination, and phosphorylation plays a pivotal role during tau aggregation (23, 80).

The SUMOylation pathway seems to be involved in AD, although the precise molecular mechanism must be further elucidated. The establishment of a mouse model for AD which expresses tagged versions of SUMO isoforms would enable unbiased proteomic studies in a physiological context and would help to understand the global role of SUMO in the disease pathogenesis.

### Parkinson's disease.

Like tau, α-synuclein is a natively unfolded protein that is subject to protein modification by SUMO and is an important constituent of Lewy bodies. Lewy bodies are inclusions of proteins, including α-synuclein, which are hallmarks of Parkinson's disease (PD) and other so-called synucleinopathies, like dementia with Lewy bodies (DLB) and multiple system atrophy (MSA) (23, 66). In contrast to tau where SUMOylation was shown to promote aggregation, SUMOylation of α-synuclein seems to increase solubility and protects against the cytotoxic effect of α-synuclein inclusions (1, 66).

Other proteins proposed to contribute to the pathogenesis of PD were reported to be SUMOylated. DJ-1, a multifunctional protein with a role in cytoprotection upon UV and oxidative stress, is strongly expressed in reactive astrocytes of patients with a sporadic form of PD and notably also in astrocytes adjacent to brain infarct regions (9, 88). SUMO1 can modify DJ-1 at K130 and consequently stimulate cell transformation and growth (115). Mutagenesis of the K130 abolished DJ-1 function. Additionally, it was shown that a mutant form of DJ-1, found in a small subset of PD patients, was improperly SUMOylated, which led to insolubility of DJ-1 (108). This shows that SUMOylation cannot only lead to an increased solubility of its targets, but SUMOylation at different sites can have opposite effects.

Parkin, an E3 ubiquitin ligase mutated in many cases of early-onset PD and immune-reactive with Lewy bodies in other forms of PD, was shown to selectively bind to SUMO1 (63, 102, 123). This interaction stimulated Parkin's translocation to the nucleus and its proteasomal degradation through an enhanced autoubiquitination (123). Whether the interaction of SUMO and Parkin promotes or impedes cell survival is not yet completely understood as Parkin targets misfolded proteins and is involved in multiple processes dependent on its localization.

Parkin as well as SUMOylation seem to be involved in mitochondrial fusion and fission, processes with particular importance in brain cells and neurodegeneration. Here, it was shown that the dynamine-related protein 1 (Drp1) is a target for conjugation by SUMO1, SUMO2, and SUMO3 (30). The modification of Drp1 by SUMO1 led to an increased mitochondrial fission, and SENP5 was shown to be the responsible SUMO protease in this process (150). Drp1 is also a reported target of Parkin, which is responsible for Drp1 ubiquitination and subsequent degradation (129). SUMOylation is thus involved in multiple processes of PD pathogenesis, and complex interplay between SUMO targets complicates the prediction of overall contribution of SUMOylation (reviewed by ref. 26).

### Polyglutamine disorders.

Protein aggregation and disruption of protein homeostasis is a common feature of polyglutamine disorders, characterized by the CAG repeat extension of genes which influences the gain of toxicity of their protein counterparts and their loss of normal function (69). These diseases include Huntington's disease (HD), spinobulbar muscular atrophy (SBMA), dentatorubral-pallidoluysian atrophy (DRPLA), and spinocerebellar ataxias (SCA). SUMOylation of the toxic proteins appears to reduce their aggregation potential (Table 1) (55, 86, 109, 120).

In Huntington's disease (HD), however, it is debated if the formation of inclusions of the causative mutant huntingtin protein (mHTT) is neurotoxic, with strong indications that this is a neuroprotective event (5, 68, 100). Even though mHTT is found to be expressed in different cell types throughout the body, degeneration is restricted to the brain's corpus striatum. A proposed explanation for this observation is the presence of the E3 SUMO ligase Rhes in the corpus striatum. Rhes was able to stimulate the SUMOylation of mHTT, but not the wild type, and mHTT SUMOylation led to a decrease in aggregate formation and an increase of cytotoxicity in vitro and in vivo (109, 112). Rhes is an unusual SUMO E3 ligase, which shows no structural similarity with other SUMO E3s, but was shown to enhance cross-SUMOylation between the SUMO E1 and UBC9 as well as thioester transfer from E1 to Ubc9 (111).

The observation that mHTT is subject to modification by SUMO and that even though SUMOylation reduces aggregates, it has a cytotoxic rather than a cytoprotective role, illustrates that SUMOylation has diverse consequences on different disease-associated proteins in distinct neurodegenerative diseases.

Taken together, these neurodegenerative diseases, discussed above, all show the accumulation of proteins into aggregates and the involvement of SUMO in the regulation of this processes. It is, however, debatable whether those aggregates are causal or consequential, neurotoxic, or even neuroprotective. In addition, the contribution of SUMO seems to have diverse effects on different aggregates and various cellular outcomes which precludes attributing a general consequence to SUMOylation (Table 1). The range of mechanisms employed by SUMO to regulate the homeostasis of proteins is broad. The examples reviewed above show that SUMO can negatively affect protein aggregation, which is consistent with the findings that the fusion of SUMO to a protein enhances their solubility and is therefore often used as a method to produce recombinant proteins in *Escherichia coli* (81). On the other hand, SUMOylation can stimulate the formation of protein complexes. Because of the existence of SIMs on proteins, which have a binding affinity to SUMO moieties attached to another protein, SUMO can stimulate complex formation. These SUMO-SIM interactions within a complex are proposed to be redundant, indicating that the overall SUMOylation status of protein complexes might be more important than SUMOylation of a single group member (reviewed by ref. 57).

## SUMO Plays a Protective Role in Brain Ischemia

In addition to SUMO's upcoming role in neurodegenerative diseases, the role of SUMO is explored in other brain-related diseases which are connected to the distortion of proteostasis. Brain ischemia is characterized by a restriction of blood supply to a region of the brain, leading to oxygen and nutrient deprivation of cells. This shortage of supplies results in the damaging of macromolecules and a general imbalance of proteostasis including reduced production of new proteins which can ultimately lead to cell death of neurons and severe brain damage (135). Natural resistance against ischemia can be observed in hibernating animals. Interestingly, it was observed that SUMO2/3 conjugation is massively increased in brains of hibernating thirteen-lined ground squirrels (*Ictidomys tridecemlineatus*) during torpor (76). Consistently, multiple studies showed a high increase of SUMO conjugation after transient ischemia in vitro and in vivo (18, 76, 79, 131, 142, 143). This raises the intriguing question about the functional contribution of SUMOylation to protect cells from damage during ischemia. Below, we review multiple studies aiming to answer this question.

In vitro the consequences of SUMOylation during ischemia were studied using the oxygen-glucose deprivation (OGD) model in combination with a neuroblastoma cell line or in primary neurons isolated from mice or rats (17, 49). By using these models, it was shown that stimulating SUMOylation by overexpression of UBC9, SUMO1, and SUMO2 increased the resistance of cells towards OGD (74, 76). Consistently, decreasing global SUMOylation by expressing a dominant negative mutant of UBC9, silencing of each of the endogenous SUMO isoforms, or the overexpression of the sentrin-specific peptidase 1 (SENP1) caused sensitization of the cells towards OGD (17, 20, 74, 76). These findings all argue for a cytoprotective role of SUMOylation during OGD in vitro.

In vivo it was shown that the overexpression of UBC9 in mice subjected to focal cerebral ischemia contributed to the protection against brain damage, as infarct size inversely correlated with the level of UBC9 overexpression (77). Further investigation of SUMOs potential neuroprotective role against ischemic damage in vivo is hampered by the challenges of establishing conditional knockout mice of the SUMO-conjugating machinery, since the SUMO E1 and E2 enzymes are essential for embryonic development. Also the differential roles of the SUMO isoforms are difficult to establish in vivo because of the redundancy of the SUMO isoforms SUMO1 and SUMO3.

### In vivo proteomics reveals SUMOylation targets on ischemia.

A different strategy to obtain insight in the role of SUMOylation in ischemia is the identification of SUMOylated proteins. Recent advances in proteomics and the generation of mice expressing tagged versions of the SUMO isoforms enabled the identification of the SUMO3 proteome in mice subjected to ischemia (141).

The proteomic data suggest a global upregulation of crosstalk between SUMO and ubiquitin during ischemia, as many proteins are shown to be targets for both modifications on different lysines. Upon silencing of SUMO2 and 3, a decrease in ubiquitin conjugation in response to ischemia is detected. This observation implies a pivotal role for SUMO-dependent ubiquitination, which is regulated by SUMO-targeted ubiquitin ligases (STUbL) and reflects the involvement of SUMO in protein homeostasis (141).

Additionally, SUMO targets were enriched for proteins involved in posttranscriptional modification of RNA. Notably, after heat shock, a distinct kind of proteotoxic stress, a large group of targets increased for SUMOylation play a role in RNA posttranscriptional modification. Moreover, SUMOylated proteins were shown to be recruited to promoters of genes involved in RNA processing (35, 92, 107). Taken together, those results indicate that SUMOylation might be involved, on multiple levels, in global regulation of RNA processes after different proteotoxic stresses.

Although the effect of SUMOylation for a single target protein is sometimes challenging to identify, SUMOylation of several important SUMO targets could contribute to the survival of neurons after deprivation of oxygen and nutrients. One of the major SUMOylation targets after ischemic stress, identified in the proteomic study by Paschen and coworkers, is the glucocorticoid receptor (GR) (141). The increased activity of GR, during chronic stress, was shown to increase the size of brain lesions after transient ischemia (8). GR SUMOylation leads to repression of its transcriptional activity and could therefore be involved in promoting cell survival (21). However, whether SUMOylation of GR directly contributes to the observed protective effect of global SUMOylation after ischemia is still unclear.

### Other ubiquitin-like proteins are highly expressed on ischemia.

SUMO was not the only UBL that was upregulated during torpor in hibernating squirrels. Protein modification by ISG15, NEDD8, UFM1, and FUB1, but interestingly not ubiquitin, was increased, which suggests that posttranslational modifications are an important regulatory mechanism when energy is scarce. Possibly, this regulation facilitates the intriguing natural tolerance against ischemic conditions observed in hibernating animals (75).

The global upregulation of UBL conjugation involves two families of microRNAs, miR-200 and mi-R182, which were downregulated during hibernation torpor. Inhibiting these miRNAs led to an increase in UBL conjugation while overexpression led to a decrease in conjugates, suggesting an important role of miRNAs in regulation of UBL conjugation (75).

In conclusion, SUMOylation is suggested as a global protective mechanism against the damaging effects of oxygen and nutrient deprivation. Because of the vast bio-complexity that underlies brain injuries due to ischemia, SUMOylation with its broad variety of target proteins would be an attractive drug target. Stimulating SUMOylation could potentially have a positive effect on neuronal cell survival. Conversely, inhibition of SUMOylation was indicated to potentially promote cell death of cancer cells. The role of the SUMOylation machinery and SUMOylation targets in cancer cells was recently reviewed by Eifler and Vertegaal (27).

## Conclusion and Future Perspectives

SUMO can either alone or in cooperation with ubiquitin regulate proteostasis in the cell. Independently and directly, SUMO is involved in the regulation of protein aggregation or solubility in neurodegenerative diseases (Table 1). The consequences of SUMOylation regarding solubility of proteins are diverse and requires additional investigation. Possibly, SUMO regulates multiple processes in those diseases and the overall cellular outcome of SUMOylation might be difficult to predict. For example, the SUMOylation of the polyQ androgen receptor (AR) was shown to decrease aggregation, but on the other hand, inhibiting SUMO increased AR transcriptional activity and ameliorated harmful properties of polyQ AR (16, 86). Combining the existing in vivo mouse models expressing tagged versions of SUMO (122, 141) with neurodegenerative disease mouse models would allow a global identification of SUMO conjugates. This would improve the understanding of SUMO's contribution to the diseases with SUMO cycle enzymes as potential drug targets (122, 141).

In addition to the direct effect of SUMOylation on proteostasis, SUMO can also influence proteostasis indirectly. One example is the regulation of transcription factors by SUMOylation, which can alter expression levels of proteins involved in maintaining proteostasis (89, 92, 107).

**Fig. 2.**
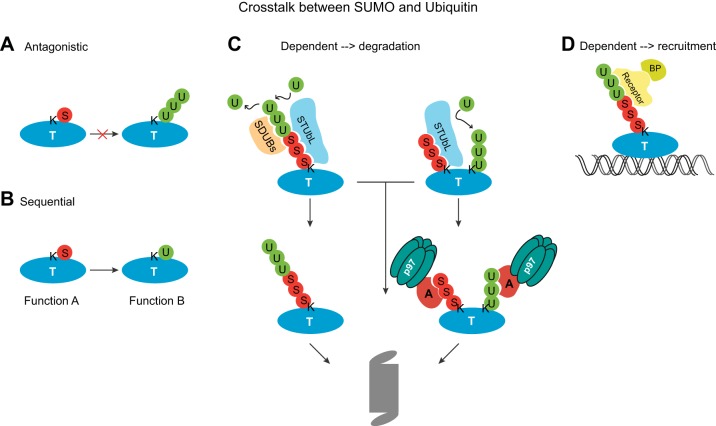
Crosstalk between ubiquitin and SUMO. SUMO and ubiquitin can influence one another in multiple ways as detailed below. *A*: antagonistic. SUMO (S) and ubiquitin (U) can compete for the modification of acceptor lysines (K). SUMOylation could thereby antagonize the ubiquitination and subsequent degradation of a target protein (T). *B*: sequential. SUMO and ubiquitin can modify the same lysine, thereby cooperatively controlling different functions of the target protein in space and time. *C*: dependent-degradation. SUMO modification of a target protein can recruit a SUMO-targeted ubiquitin ligase (STUbL), which is able to ubiquitinate the protein either on the existing SUMO chain or on a different acceptor lysine. The ubiquitin chains can either direct the protein to the proteasome for degradation or recruit the CDC48/p97 chaperone via its adaptor proteins (A), which removes the ubiquitinated protein from the chromatin and delivers it to the proteasome. SUMO deubiquitinases (SDUBs) can reverse the action of STUbLs and remove ubiquitin from SUMOylated targets, thereby stabilizing the protein. *D*: dependent recruitment. Hybrid chains of SUMO and ubiquitin generated by STUbLs can serve as recruitment platforms for receptors and binding partners (BP) at the chromatin.

The main contribution of SUMO in protein homeostasis is, however, its complex interplay with ubiquitin (Fig. 2). The ubiquitin-proteasome system (UPS) is involved in multiple signaling pathways and plays a major role in quality control of proteins (34). After synthesis, around 30% of proteins are misfolded and need to be degraded to prevent a constitutive unfolded protein response and subsequent apoptosis (104). It is critical that protein quality control is tightly regulated. SUMOylation was shown to either inhibit or promote ubiquitination of targets and their subsequent degradation and therefore is a component of the tightly controlled UPS. The process of SUMO-dependent ubiquitination is being extensively explored, leading to the identification of enzymes involved in this crosstalk, like STUbLs and SUMO-targeted deubiquitinases. In mammals, two enzymes of each class have been identified (45, 72, 113, 118). Proteins that are misfolded or unfolded during stress are ubiquitinated and targeted to the proteasome. It was found that those stre sses also strongly induce SUMOylation of targets (44, 101, 119). Yet, it is unclear why and how this increase in SUMOylation upon heat stress or inhibition of the proteasome takes place, but it cannot only be explained by the accumulation of SUMO-conjugates. The possibility that SUMO is actively conjugated to ubiquitin targets opens a whole new area to be explored. Do ubiquitin-targeted SUMO ligases exist? Which consequence has the SUMOylation of ubiquitinated proteins? Does this involve the ability of SUMO to alter solubility and therefore stimulates cell survival by giving the UPS time to catch up with the load of misfolded proteins? These questions need to be answered in the future and could lead to the exploration of SUMO enzymes as potential drug targets in diseases that are characterized by an unbalanced proteostasis, including neurodegenerative diseases.

## GRANTS

The authors are grateful for support from the European Research Council, Grant 310913 (A.C.O. Vertegaal).

## DISCLOSURES

No conflicts of interest, financial or otherwise, are declared by the author(s).

## AUTHOR CONTRIBUTIONS

F.L. prepared figures; F.L. drafted manuscript; F.L. and A.C.O.V. approved final version of manuscript; A.C.O.V. edited and revised manuscript.

## Glossary

AD: Alzheimer's disease
Aβ: Amyloid-β
AR: Androgen receptor
APP: Amyloid precursor protein
ATO: Arsenic trioxide
Axin: Axis Inhibition protein 1
BRCA1-A: Breast cancer type 1 susceptibility protein 1-A
CDC48: Cell division control protein 48
c-Ski: v-ski avian sarcoma viral oncogene homolog
c-Myc: v-myc avian myelocytomatosis viral oncogene homolog
DeSI: DeSumoylating isopeptidase
DJ-1: Protein deglycase DJ-1
DLB: Dementia with Lewy bodies
Drp1: Dynamine-related protein 1
DRPLA: Dentatorubral-pallidoluysian atrophy
DVC: DNA damage protein targeting VCP
FANCI: Fanconi anemia group I
FANCD2: Fanconi anemia group D 2
FUBI: Finkel-Biskis-Reilly murine sarcoma virus (FBR-MuSV) ubiquitously expressed
GR: Glucocorticoid receptor
HD: Huntington's disease
HS: Heat shock
HSP: Heat shock protein
IκB-α: Inhibitor of nuclear factor-κB
ISG15: Interferon-induced 15-kDa protein
MAP: Mitogen-activated protein
mHTT: Mutant Huntingtin protein
miRNA: microRNA
MS: Mass spectrometry
MSA: Multiple system atrophy
NEDD8: Neural precursor cell expressed developmentally downregulated protein 8
NEMO: Nuclear factor-κB essential modulator
Nlp4: Nuclear localization protein 4 homolog
OGD: Oxygen-glucose deprivation
p14/ARF: Tumor suppressor ARF
PC2/CBX4: Polycomb 2 homolog/chromobox protein homolog 4
PCNA: Proliferating cell nuclear antigen
PD: Parkinson's disease
PIAS: Protein inhibitor of activated STAT
PML: Promyelocytic leukemia
PN: Proteostasis network
RAD18: E3 ubiquitin ligase RAD18
RanBP2: RAN binding protein 2
RAP80: Receptor-associated protein 80
Rhes: Ras homolog enriched in striatum
RNF4: Ring-finger protein 4
RNF111: Ring-finger protein 111
RSUME: RWD-containing SUMOylation enhancer
SBMA: Spinobulbar muscular atrophy
SCA: Spinocerebellar ataxis
SAE1: SUMO-activating enzyme subunit 1
SAE2: SUMO-activating enzyme subunit 2
SDUB: SUMO deubiquitinase
SENP: Sentrin-specific protease
SHMT1: Serine hydroxymethyltransferase 1
SLX4: Structure-specific endonuclease subunit
SMAD: Mothers against decapentaplegic homolog
SnoN: SKI-like protein
STAT: Signal transducer and activator of transcription
STUbL: SUMO-targeted ubiquitin ligase
SUMO: Small ubiquitin-like modifier
tau: Microtubule-associated protein tau
TGF-β: Transforming growth factor-β
Topors: Topoisomerase I binding, arginine/serine-rich, E3 ubiquitin protein ligase
UBL: Ubiquitin-like proteins
UBA2: Ubiquitin-like modifier-activating enzyme 2
UBC9: SUMO-conjugating enzyme UBC9
Ufd1: Ubiquitin fusion degradation protein 1 homolog
UFM1: Ubiquitin-fold modifier 1
UIM: Ubiquitin-interaction motif
Ulp: UBL-specific protease
ULS: Ubiquitin ligase for SUMOylation
UPS: Ubiquitin proteasome system
USP7: Ubiquitin-specific processing protease 7
